# Association of red blood cell distribution width–albumin ratio with in-hospital mortality in abdominal aortic aneurysm patients

**DOI:** 10.1097/MD.0000000000040785

**Published:** 2024-12-06

**Authors:** Chao Weng, Cong Yu, Guang-Wei Yang, Jin-Song Jiang, Hao Wu

**Affiliations:** a Department of Vascular Surgery, General Surgery, Cancer Center, Zhejiang Provincial People’s Hospital, Affiliated People’s Hospital, Hangzhou Medical College, Hangzhou, Zhejiang, China.

**Keywords:** abdominal aortic aneurysm, in-hospital mortality, prognosis, red blood cell distribution width–albumin ratio

## Abstract

To explore whether red blood cell distribution width–albumin ratio (RAR) is relevant to in-hospital mortality among abdominal aortic aneurysm (AAA). This is a retrospective study retrieving data from the MIMIC-IV database. Patients were divided into survivor or non-survivor groups by the in-hospital mortality. Receiver operating characteristic curve analysis, logistic regression models, subgroup analysis, interaction analysis, and restricted cubic spline analysis were conducted to analyze the correlation between RAR and in-hospital mortality. Then, we divided patients into 2 groups by an optimal cutoff value of RAR to identify the factors independently linked to RAR. Following this, the mediation analysis was conducted to reveal the potential regulatory path. Finally, we assessed the clinical value of RAR in secondary outcomes containing length of hospital stay, intensive care unit (ICU) admission, and ICU stay. Totally 770 participants with AAA were enrolled: 722 survivors and 48 non-survivors. Higher RAR was observed in the non-survivor group and its level performed satisfactorily in predicting in-hospital mortality. AAA patients were more likely to die during in-hospital with the increase of RAR (*P* < .05) and this linear correlation was revealed by restricted cubic spline (*P* non-linear > .05). Additionally, urea nitrogen and creatinine were independently related to RAR. RAR served as a mediator in the association of urea nitrogen/creatinine with in-hospital mortality. Finally, the length of hospital stay and ICU stay were longer in the RAR ≥ 4.658 group (*P* < .05). RAR is a potential risk predictor for in-hospital mortality in AAA patients. Further, RAR upregulation was significantly correlated with prolonged length of hospital stay and ICU stay.

## 1. Introduction

Abdominal aortic aneurysm (AAA) is a cardiovascular disease defined as a maximum aortic diameter of ≥30 mm on ultrasonography or computed tomography imaging.^[1]^ Oxidative stress and inflammation play a major role in the etiology of AAA.^[2]^ Most patients with an AAA have no symptoms unless complications occur including AAA ruptures which are fatal.^[3]^ The indication for AAA treatment (endovascular aortic repair or open surgical repair) includes an aneurysm diameter >5 cm in females, >5.5 cm in males, or rates of growth of >0.5 cm in 6 months or >1 cm in 1 year.^[4]^ Male sex, older age, tobacco use, hypertension, atherosclerosis, and genetics are the main risk factors for AAA.^[5]^ The prevalence rate of AAA in men is higher than in women (4:1 ratio); however, women have an accelerated rate of aneurysm growth.^[6]^ Besides, among quitters, the risk for AAA at 25 years after smoking cessation was similar to that of never-smokers.^[7]^ The prevalence of AAA in men over 65 years old has decreased from >5% to 1 to 2% in developed countries, mainly due to a decline in smoking rates.^[8,9]^ Interestingly, the prevalence of AAA in developing countries is lower than in developed countries, which may result from under-diagnosis in developing countries rather than a true lower incidence.^[10]^ Approximately 167,200 patients die from AAA worldwide per year, and there are about 15,000 deaths in the United States annually.^[11]^ Therefore, it is necessary to investigate the modifiable factors for improving the prognosis of AAA.

Red blood cell distribution width (RDW) is an easily obtained hematological parameter, that quantifies red blood cell (RBC) size in peripheral circulation and reflects the heterogeneity of erythrocyte volume.^[12]^ RDW is traditionally used for the differential diagnosis of anemia.^[13]^ The normal reference range of RDW in the general population is 11% to 15%, and its greater value is closely associated with a larger difference in RBC shape and size.^[14]^ Besides, elevated RDW levels reflect an imbalance of RBC homeostasis and impaired RBC growth. This may be related to oxidative stress, vascular inflammation, and nutritional deficiency (e.g., folic acid, vitamin B, and iron).^[15]^ As a biochemical marker of nutritional and inflammatory status, albumin (ALB), the main substance to maintain plasma osmotic pressure, is synthesized in the liver.^[16]^ ALB exerts a neuroprotective role through anti-inflammatory activity, and antioxidant characteristics, inhibiting endothelial cell apoptosis, and regulating microvascular permeability.^[17,18]^ The RDW/ALB ratio (RAR) is a new combined biomarker based on RDW and ALB. A previous study has revealed that the RAR had a more satisfactory performance in predicting stroke than RDW.^[19]^ In younger and less severely ill patients with diabetic foot ulcers, the ability of RAR was superior to RDW in predicting mortality.^[20]^ Additionally, the value of RAR for predicting mortality in patients with diabetic retinopathy has been reported.^[15]^ RAR also has a good predictive effect on death in patients with burn surgery and diabetic ketoacidosis.^[21]^ Overall, RAR can better predict inflammation-related diseases than RDW. Nevertheless, few research investigated the predictive value of RAR in the prognosis of AAA patients.

In addition to analyzing the association of RAR with in-hospital mortality in AAA subjects, we also assessed the clinical significance of RAR in length of hospital stay, intensive care unit (ICU) admission, and ICU stay.

## 2. Materials and methods

### 2.1. Data source

The study employed a retrospective design on basis of data from MIMIC-IV. This database is publicly available consisting of information on patients admitted to Beth Israel Deaconess Medical Center from 2008 to 2019. Personal information was thoroughly protected. Therefore, the informed consent of patients was not applicable. This study was approved by the Massachusetts Institute of Technology and the Institutional Review Boards.

### 2.2. Study population

Patients diagnosed with AAA are based on the international classification of disease codes-10 (ICD-10) (I713 and I714). Inclusion criteria: AAA patients aged over 18 years old; length of hospital stay ≥48 hours; with RDW and ALB data.

### 2.3. Data extraction

The extracted data included demographic information, lifestyle, comorbidities, initial laboratory findings within 24 hours, and prognostic scoring systems by the Navicat Premium (version 15.0) with Structured Query Language programming. Demographics include age, gender, body mass index (BMI), and marital status. Lifestyle includes smoking and alcohol use. Comorbidities include chronic obstructive pulmonary disease (COPD), hyperlipidemia, coronary heart disease (CHD), and hypertension. Laboratory parameters include lymphocytes, neutrophils, platelets, urea nitrogen, creatinine, anion gap, lactate, RDW, ALB, and RAR. Prognostic scoring systems include SOFA and APSIII. In addition, the ruptured state of AAA and surgery type were also collected. We adopted data from the first hospitalization of patients who were admitted more than once.

In-hospital mortality was used as the primary outcome. Length of hospital stay, ICU admission, and ICU stay were considered as the secondary outcomes.

### 2.4. Statistical analysis

Patients were divided into survivor and non-survivor groups in accordance with the in-hospital mortality. We presented categorical variables as count (percent) and used the Chi-square test or Fisher exact test for group comparisons. As for continuous variables, the Shapiro–Wilk test was employed to check the normal distribution. Continuous variables were presented as mean ± standard deviation (normal distribution) or medians (quartiles) (skewed distribution), and the differences were compared by the independent *t* test or the Mann–Whitney *U* tests, respectively.

To determine the predictive value of RAR for in-hospital mortality, we performed the receiver operating characteristic (ROC) curve analysis and obtained the optimal cutoff value of RAR with the best performance. Delong test was used to analyze the statistical differences in the area under the curve (AUCs). Following this, logistic regression models were used to analyze the association of RAR as a continuous variable with in-hospital mortality. The variables that were considered clinically relevant were enrolled for multivariable regression analysis reported by Stone et al. Given the number of available events, the variables included were carefully selected to ensure parsimony of the final model.^[22]^ Clinically relevant variables include demographics, lifestyle factors, comorbidities, and laboratory parameters. Therefore, except for RAR, multivariable model 1: age, gender, BMI, marital status, smoking, and alcohol use were adjusted; multivariable model 2: COPD, CHD, hyperlipidemia, and hypertension were adjusted; multivariable model 3: lymphocytes, neutrophils, platelets, urea nitrogen, creatinine, anion gap, and lactate were adjusted; multivariable model 4: rupture state of AAA and surgery type were adjusted. Besides, RAR was transformed into a categorical variable according to the cutoff value and quartiles to further investigate its value in predicting in-hospital mortality for sensitivity analysis to test the model’s robustness. The correlation was further conducted in different subgroups according to BMI (<18.5/18.5–23.9/24.0–27.9/≥28.0), gender (female/male), hypertension (no/yes), hyperlipidemia (no/yes), CHD (no/yes), and COPD (no/yes). The interaction analysis was undertaken to examine the interactors of the correlation between RAR and in-hospital mortality. The nonlinearity of this correlation was determined using the restricted cubic spline method.

Moreover, we categorized enrolled participants into the RAR < 4.658 and RAR ≥ 4.658 groups based on the cutoff value. Next, we explored the independent risk factors for predicting RAR. Finally, the mediation analysis was performed to explore the potential regulatory path. All analyses were performed using R software (version 4.1.2), Navicat Premium (version 15.0), and SPSS software (version 23.0) and a *P*-value < .05 indicates statistical significance.

## 3. Results

### 3.1. Characteristics of participants

There were 770 participants meeting the inclusion criteria. Subjects in the non-survivor group (n = 48) were likely to be older than the survivor group (n = 722) with a median age of 78.00 years versus 75.00 years, but accounting a higher proportion of non-hyperlipidemia (*P* < .05) (Table 1). Besides, significantly lower lymphocytes, and ALB, but had notably higher neutrophils, urea nitrogen, creatinine, anion gap, lactate, RDW, and RAR were found in the non-survivor group (*P* < .05). In addition, SOFA and APSIII scores in the non-survivor group were remarkably higher (*P* < .05). Length of hospital stay and ICU stay in the non-survivor group were also notably longer with statistical significance (*P* < .05). The proportion of AAA rupture in the non-survivor group was significantly higher than that in the survival group (*P* < .05). However, other variables including BMI, gender, smoking, alcohol use, marital status, surgery type, COPD, CHD, hypertension, platelet, and ICU admission made no difference to mortality (all *P* > .05) (Table 1).

**Table 1:** Patient characteristics.

Characteristics	Survivor (n = 722)	Non-survivor (n = 48)	*P*-value
Age (years)	75.00 [68.00, 82.00]	78.00 [74.00, 83.00]	.027
Body mass index (kg/m^2^)	26.00 [22.60, 29.50]	25.30 [21.40, 27.90]	.254
Gender, n (%)			.100
Male	503 (69.67)	28 (58.33)	
Female	219 (30.33)	20 (41.67)	
Smoking, n (%)			.803
No	654 (90.58)	44 (91.67)	
Yes	68 (9.42)	4 (8.33)	
Alcohol use, n (%)			.437
No	690 (95.57)	47 (97.92)	
Yes	32 (4.43)	1 (2.08)	
Marital status, n (%)			.529
Single	134 (18.56)	7 (14.58)	
Married	362 (50.14)	27 (56.25)	
Divorced/widowed	190 (26.32)	10 (20.84)	
Unknown	36 (4.98)	4 (8.33)	
Ruptured state, n (%)			<.001
No	699 (96.81)	41 (85.42)	
Yes	23 (3.19)	7 (14.58)	
Surgery type, n (%)			.237
No surgery	39 (81.25)	506 (70.08)	
Endovascular surgery	7 (14.58)	151 (20.91)	
Open surgery	2 (4.17)	65 (9.01)	
COPD, n (%)			.974
No	528 (73.13)	35 (72.92)	
Yes	194 (26.87)	13 (27.08)	
Hyperlipidemia, n (%)			.036
No	295 (40.86)	27 (56.25)	
Yes	427 (59.14)	21 (43.75)	
Coronary heart disease, n (%)			.682
No	383 (53.05)	24 (50.00)	
Yes	339 (46.95)	24 (50.00)	
Hypertension, n (%)			.940
No	372 (51.52)	25 (52.08)	
Yes	350 (48.48)	23 (47.92)	
Lymphocyte (%)	14.80 [7.90, 21.80]	8.30 [4.50, 13.30]	.012
Neutrophil (%)	74.10 [65.60, 81.90]	82.40 [71.90, 86.00]	.046
Platelet counts (10^9^/L)	178.00 [136.00, 228.00]	163.00 [106.00, 240.00]	.264
Urea nitrogen (mg/dL)	20.00 [15.00, 28.00]	25.00 [16.00, 37.00]	.007
Creatinine (mg/dL)	1.00 [0.80, 1.00]	1.20 [0.90, 1.80]	.010
Anion gap (mmol/L)	14.00 [12.00, 16.00]	15.00 [12.00, 19.00]	.009
Lactate (mmol/L)	1.50 [1.10, 2.20]	1.80 [1.50, 2.90]	.044
RDW (%)	14.40 [13.30, 15.50]	15.70 [14.20, 17.60]	<.001
Albumin (g/dL)	3.50 [3.19, 3.80]	3.10 [2.73, 3.38]	<.001
RAR	4.05 [3.67, 4.84]	5.23 [4.33, 6.48]	<.001
SOFA score	4.00 [2.00, 6.00]	8.00 [5.00, 11.00]	<.001
APSIII score	39.00 [29.00, 52.00]	71.00 [55.00, 96.00]	<.001
Length of hospital stay (days)	5.37 [3.00, 8.78]	9.62 [4.81, 15.82]	<.001
ICU admission, n (%)			.652
No	267 (52.67)	143 (54.37)	
Yes	240 (47.33)	120 (45.63)	
ICU stay (days)	2.21 [1.22, 3.83]	4.23 [2.31, 9.78]	<.001

COPD = chronic obstructive pulmonary disease, ICU = intensive care unit, RAR = red blood cell distribution width/albumin ratio, RDW = red blood cell distribution width.

### 3.2. Predictive value of RAR for in-hospital mortality

**Figure 1.**
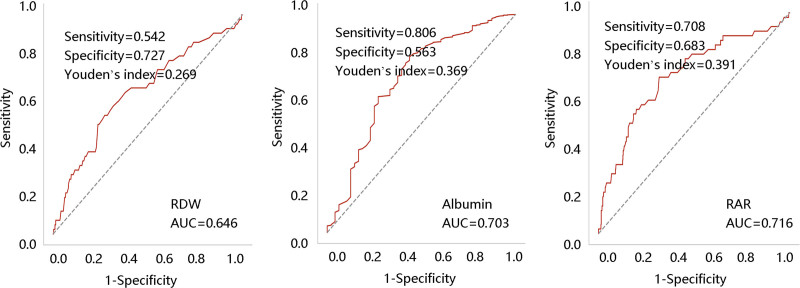
Receiver operating characteristic analysis assessed the performance of RDW, albumin, and RAR in predicting in-hospital mortality. Delong test: RAR vs RDW, *P* = .033; RAR vs albumin, *P* < .001; RDW vs albumin, *P* < .001. RAR = red blood cell distribution width/albumin ratio; RDW = red blood cell distribution width.

The ROC curves were used to compare the value of RAR, RDW, and ALB in predicting in-hospital mortality. The AUC values of RAR, RDW, and ALB were 0.716, 0.646, and 0.703, respectively, indicating the superior ability of RAR in distinguishing patients from mortality. The Delong test showed that the differences were statistically significant (*P* < .05) (Fig. 1). Of note, the cutoff value of RAR was 4.658 (Table 2).

**Table 2:** ROC analysis for the value of RAR in predicting in-hospital mortality.

Variables	Cutoff	Sensitivity	Specificity	AUC (95% confidence interval)
RDW	15.350	0.542	0.727	0.646 (0.560–0.732)
Albumin	3.125	0.563	0.806	0.703 (0.617–0.789)
RAR	4.658	0.708	0.683	0.716 (0.634–0.798)

AUC = the area under the curve, RAR = red blood cell distribution width/albumin ratio, RDW = red blood cell distribution width.

Then, logistic regression analysis was performed for in-hospital mortality. In the univariate analysis, RAR as a both continuous and categorical variable was significantly related to in-hospital mortality (*P* < .05). After adjusting demographic information and lifestyle in multivariable model 1, RAR remained a significant indicator for predicting in-hospital mortality (*P* < .05). Comorbidities were adjusted in the multivariable model 2, high RAR was significantly associated with a higher risk of in-hospital mortality (*P* < .05). Laboratory parameters were adjusted in the multivariable model 3, high RAR was significantly associated with a higher risk of in-hospital mortality (*P* < .05). To consider the influence of surgery type and rupture state on their relationship, we adjusted the 2 covariates in multivariable model 4, and the results showed that their relationship was not affected (*P* < .05). *P* for trend test showed that in-hospital mortality tended to be upregulated with the elevation of RAR (*P* for trend < .01) (Table 3).

**Table 3:** The association of RAR with in-hospital mortality using logistic regression analysis.

Variables	Univariate model		Multivariate model 1		Multivariate model 2		Multivariate model 3		Multivariate model 4	
	OR (95% CI)	*P*-value	OR (95% CI)	*P*-value	OR (95% CI)	*P*-value	OR (95% CI)	*P*-value	OR (95% CI)	*P*-value
RAR	1.563 (1.325–1.842)	<.001	1.579 (1.256–1.984)	<.001	1.588 (1.342–1.879)	<.001	1.606 (1.187–2.173)	.002	1.496 (1.263–1.772)	<.001
RAR cutoff	5.228 (2.752–9.934)	<.001	4.099 (1.9928.434)	<.001	5.333 (2.785–10.214)	<.001	9.702 (2.773–33.939)	<.001	4.518 (2.357–8.662)	<.001
RAR quartiles										
Q1	Reference		Reference		Reference		Reference		Reference	
Q2	0.792 (0.209–2.994)	.731	0.894 (0.218–3.66)	.876	0.971 (0.275–3.424)	.963	2.584 (0.197–33.986)	.470	1.006 (0.284–3.560)	.992
Q3	2.273 (0.774–6.671)	.135	2.175 (0.662–7.141)	.200	2.231 (0.744–6.687)	.152	7.112 (0.736–68.754)	.090	2.16 (0.718–6.498)	.171
Q4	6.347 (2.396–16.815)	<.001	4.495 (4.493–4.496)	.009	6.579 (2.455–17.631)	<.001	16.063 (1.812–142.387)	.013	5.318 (1.984–14.252)	.001
*P* for trend		<.001		.001		<.001		.002		<.001

95% CI = 95% confidence interval, OR = odds ratio, RAR = red blood cell distribution width/albumin ratio.

**Figure 2.**
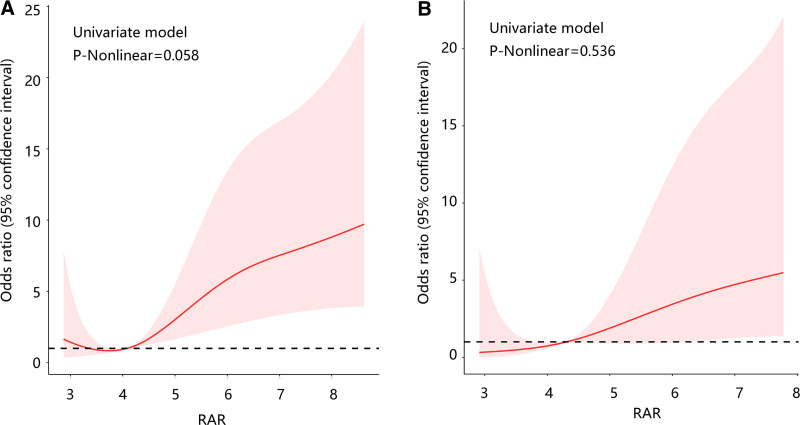
Restricted cubic spline analysis revealed the linear relationship between RAR and in-hospital mortality in 2 models. (A) Model a: no variable was adjusted. (B) Model b: age, hyperlipidemia, urea nitrogen, creatinine, anion gap, and lactate were adjusted. RAR = red blood cell distribution width/albumin ratio.

Subsequently, the subgroup analysis results showed a positive connection of RAR to in-hospital mortality among those with 24.0 ≤ BMI ≤ 27.9, and BMI ≥ 28.0 regardless of gender, hypertension, hyperlipidemia, CHD, and COPD (all *P* < .05) (Table 4). Besides, BMI, gender, hypertension, hyperlipidemia, CHD, and COPD were not interactors of this relationship (*P* for interaction > .05) (Table 4). Restricted cubic spline analysis results exhibited that there was a linear association of RAR with in-hospital mortality (*P*-non-linear > .05) (Fig. 2A and B).

**Table 4:** Subgroup analysis of the correlation between RAR and in-hospital mortality.

Subgroups	Odds ratio (95% confidence interval)	*P*-value	*P* for interaction
BMI (kg/m^2^)			.456
<18.5	3.500 (0.294–41.702)	.322	
18.5–23.9	2.137 (0.553–8.257)	.271	
24.0–27.9	5.623 (1.726–18.320)	.004	
≥28.0	5.607 (1.36–23.111)	.017	
Gender			.236
Female	5.645 (1.976–16.125)	.001	
Male	4.829 (2.137–10.916)	<.001	
Hypertension			.765
No	5.495 (2.143–14.092)	<.001	
Yes	5.181 (2.127–12.621)	<.001	
Hyperlipidemia			.214
No	4.923 (2.081–11.648)	<.001	
Yes	5.526 (2.098–14.558)	.001	
CHD			.918
No	5.66 (2.285–14.017)	<.001	
Yes	4.793 (1.932–11.891)	.001	
COPD			.835
No	5.203 (2.488–10.88)	<.001	
Yes	5.525 (1.472–20.735)	.011	

BMI = body mass index, CHD = coronary heart disease, COPD = chronic obstructive pulmonary disease.

### 3.3. Mediation analysis

As shown in Table 5, the distribution of alcohol use, COPD, hypertension, age, BMI, lymphocytes, urea nitrogen, creatinine, and lactate was significantly different between RAR < 4.658 and RAR ≥ 4.658 groups (*P* < .05), which were then included in the multivariable logistic regression analysis. Urea nitrogen and creatinine were independent indicators for predicting RAR (*P* < .05), while other factors were not significantly associated with RAR (all *P* > .05) (Table 6). Upon mediation analysis, the direct effect represented the association of creatinine and urea nitrogen with in-hospital mortality; the indirect effect represented that the association of creatinine and urea nitrogen with in-hospital mortality was mediated by RAR. The results showed that RAR partially mediated the association of creatinine with in-hospital mortality and totally mediated the association of urea nitrogen with in-hospital mortality (Table 7).

**Table 5:** The distribution of clinical parameters in 2 RAR groups.

Characteristics	RAR < 4.658 (n = 507)	RAR ≥ 4.658 (n = 263)	*P*-value
Gender, n (%)			.124
Male	359 (70.81)	172 (65.40)	
Female	148 (29.19)	91 (34.60)	
Smoking, n (%)	462 (91.12)	236 (89.73)	.530
No	45 (8.88)	27 (10.27)	
Yes			
Alcohol use, n (%)			.012
No	492 (97.04)	245 (93.16)	
Yes	15 (2.96)	18 (6.84)	
Marital status, n (%)			.119
Single	93 (18.34)	48 (18.25)	
Married	270 (53.25)	119 (45.25)	
Divorced/widowed	120 (23.67)	80 (30.42)	
Unknown	24 (4.73)	16 (6.08)	
COPD, n (%)			.035
No	383 (75.54)	180 (68.44)	
Yes	124 (24.46)	83 (31.56)	
Hyperlipidemia, n (%)			.439
No	207 (40.83)	115 (43.73)	
Yes	300 (59.17)	148 (56.27)	
CHD, n (%)			.286
No	275 (54.24)	132 (50.19)	
Yes	232 (45.76)	131 (49.81)	
Hypertension, n (%)			.003
No	242 (47.73)	155 (58.94)	
Yes	265 (52.27)	108 (41.06)	
Age (years)	75.00 [67.00, 81.00]	77.00 [70.00, 82.00]	.026
BMI (kg/m^2^)	26.30 [23.00, 30.00]	25.50 [21.90, 28.50]	.010
Lymphocyte (%)	15.70 [8.30, 22.50]	11.00 [5.80, 18.20]	.004
Neutrophil (%)	74.10 [65.10, 82.00]	76.00 [66.50, 83.70]	.366
Platelet counts (10^9^/L)	178.00 [140.00, 223.00]	172.00 [123.00, 255.00]	.681
Urea nitrogen (mg/dL	19.00 [14.00, 25.00]	23.00 [16.00, 36.00]	<.001
Creatinine (mg/dL)	1.00 [0.80, 1.30]	1.10 [0.90, 1.60]	<.001
Anion gap (mmol/L)	14.00 [12.00, 16.00]	14.00 [12.00,17.00]	.191
Lactate (mmol/L)	1.50 [1.10, 2.10]	1.70 [1.40, 2.70]	.002

BMI = body mass index, CHD = coronary heart disease, COPD = chronic obstructive pulmonary disease, RAR = red blood cell distribution width/albumin ratio.

**Table 6:** Identification of independent predictors for RAR using multivariate logistic regression analysis.

Variables	Odds ratio	95% CI-low	95% CI-high	*P*-value
Alcohol use	0.859	0.140	5.280	.870
Hypertension	0.630	0.281	1.413	.262
Age	0.989	0.955	1.024	.527
BMI	0.965	0.904	1.031	.291
Lymphocyte	0.994	0.959	1.030	.745
Urea nitrogen	1.067	1.025	1.111	.002
Creatinine	0.383	0.177	0.827	.015
Lactate	1.178	0.927	1.497	.180

95% CI = 95% confidence interval, BMI = body mass index, RAR = red blood cell distribution width/albumin ratio.

**Table 7:** The mediation role of RAR.

Path	Coef	*P*-value	Significance
RAR–creatinine	0.230	<.001	Yes
Y–RAR	0.041	<.001	Yes
Total	0.035	<.001	Yes
Direct	0.027	.002	Yes
Indirect	0.009	<.001	Yes
RAR–urea nitrogen	0.017	<.001	Yes
Y–RAR	0.041	<.001	Yes
Total	0.001	.006	Yes
Direct	0.001	.139	No
Indirect	0.001	<.001	Yes

RAR = red blood cell distribution width/albumin ratio.

### 3.4. Clinical value of RAR in secondary outcomes

To further explore the clinical value of RAR, we compared the length of hospital stay, ICU admission, and ICU stay between the low and high RAR groups based on the cutoff value of RAR. Length of hospital stay and ICU stay was longer in the high RAR group compared with those in the low RAR group with statistical significance (*P* < .05). Whereas, there was no significant difference in the distribution of ICU admission between the 2 groups (*P* > .05) (Table 8).

**Table 8:** The clinical value of RAR for secondary outcome.

Secondary outcome	RAR < 4.658 (n = 507)	RAR ≥ 4.658 (n = 263)	*P*-value
Length of hospital stay	5.08 [2.91, 8.67]	6.02 [3.79, 10.70]	<.001
ICU admission			.703
No	267 (52.67)	143 (54.37)	
Yes	240 (47.33)	120 (45.63)	
ICU stay	2.206 [1.21, 3.59]	2.92 [1.76, 5.87]	.003

ICU = intensive care unit, RAR = red blood cell distribution width/albumin ratio.

## 4. Discussion

In this study, RAR was significantly higher in the non-survivor group and its elevation served as an independent predictor for death during the hospital stay among AAA patients. In addition, RAR plays a mediating role in the correlation between creatinine/ urea nitrogen and in-hospital mortality. Moreover, RAR might lead to longer length of stay and ICU stay. These revealed that RAR might be a potential risk factor for the unfavorable prognosis of AAA patients.

Previous studies have demonstrated the role of RAR in several diseases. Sim et al conducted a study on 907 patients undergoing radical hysterectomy and identified that RAR increased the risk of transfusion and mortality in cervical cancer.^[23]^ Besides, Pan et al used data from the MIMIC database and revealed that a nomogram incorporating RAR exhibited good performance in predicting short-term mortality in patients with acute pancreatitis.^[24]^ In a retrospective review of patients in a burn ICU, higher RAR was found to be closely related to longer ICU stays and higher 90-day mortality.^[25]^ By extracting related data from the National Health and Nutrition Examination Survey website, Zhao et al exhibited that RAR could independently predict the risk of diabetic retinopathy after adjusting multiple covariates.^[15]^ The same function was also confirmed in diabetic ketoacidosis as well as an elevated risk of relevant infections.^[21]^ This is the first study to use the MIMIC data to analyze the role of RAR in the in-hospital mortality of AAA patients.

First, the ROC analysis was adopted to evaluate the superior value of RAR to RDW and ALB alone in predicting in-hospital mortality with a cutoff value of 4.658. Then, different logistic regression models examined the higher probability of death with an increase of RAR. RAR indicator based on RDW and ALB was connected with inflammation, oxidative stress, and endothelial dysfunction.^[19]^ On the one hand, chronic aortic inflammation results in aortic wall destruction, followed by the infiltration of the innate and adaptive immune cells including monocytes, dendritic cells, T cells, and B cells to the aortic wall through the intraluminal thrombus, the intimal atherosclerotic plaque, periadventitial lymph nodes, and adventitial vasa vasorum.^[26]^ These immune cells are the source of proteases, oxygen-derived free radicals, and cytokines. Among them, proteases induce disruption of elastic fibers and consequent loss of wall elasticity.^[27]^ Oxygen-derived free radicals can induce vascular smooth muscle cell apoptosis and phenotypic changes, resulting in a partial loss of matrix production and repair capacity of the media.^[28]^ Cytokines can exacerbate the inflammatory response. Taken together, these effects destructed the aortic media and promoted the occurrence and development of AAA.^[29]^ On the other hand, arterial endothelium provides a surface for thrombosis formation and critically regulates blood fluidity and homeostasis.^[30]^ Under laminar flow and high shear stress conditions, various antiplatelet and anticoagulant substances (such as nitric oxide and thrombomodulin) are generated by the healthy endothelium; however, the function would change from antithrombotic to pro-coagulant after endothelial injury through the platelets and leukocytes activation as well as the tissue factor induction.^[31]^ Therefore, the authors speculated that RAR might affect the in-hospital mortality of AAA patients via the involvement of inflammation and endothelial dysfunction.

Subsequently, we revealed that RAR served as a mediator in the correlation between creatinine/urea nitrogen and in-hospital mortality. Although there was no significant difference in ICU admission between the 2 RAR groups. Patients in the RAR-high group had a longer length of stay and ICU stay. These results revealed that RAR plays an essential role in the prognosis of AAA patients.

For strengths, this study employed ROC analysis to examine the predictive value of RAR for in-hospital mortality and obtained a cutoff value. Second, we adopted 4 multivariable models and performed a sensitivity analysis to analyze the association of RAR with in-hospital mortality. Third, the mediation analysis was utilized to reveal the potential regulatory path of the RAR for in-hospital mortality in AAA patients. For limitations, the retrospective study was only based on the data from the MIMIC database, which may result in patient selection bias. Thus, we adjusted various variables to ensure that the results were as accurate as possible. Additionally, we only monitored data once within the first 24 hours in the first admission but lack of dynamic observations for RAR. Therefore, studies in different cohorts with dynamic observations of RAR are required to validate the value of RAR in predicting in-hospital mortality among AAA patients. Besides, the anatomy of the AAA was not included in the analysis due to a lack of relevant data from the MIMIC database, and this variable should be considered in future research. Despite these drawbacks, our study provides a meaningful reference to the value of RAR in AAA.

In conclusion, RAR is a potential indicator of in-hospital mortality of AAA. Besides, RAR exhibits a better ability to predict in-hospital mortality than RDW and ALB alone. Moreover, higher RAR was significantly associated with longer hospital stay and ICU stay.

## Author contributions

**Conceptualization:** Chao Weng.

**Data curation:** Chao Weng, Cong Yu, Guang-Wei Yang, Hao Wu.

**Investigation:** Guang-Wei Yang, Hao Wu.

**Methodology:** Chao Weng, Cong Yu, Jin-Song Jiang.

**Supervision:** Hao Wu.

**Writing – original draft:** Chao Weng, Cong Yu, Guang-Wei Yang, Jin-Song Jiang.

**Writing – review & editing:** Hao Wu.
